# Supplementary material for: Analysis of PPARγ Signaling Activity in Psoriasis
Source: Int J Mol Sci. 2021 Aug 10;22(16):8603. doi: 10.3390/ijms22168603 (PMC8395241; doi:10.3390/ijms22168603)
Supplement: Supplementary file 1 [file ijms-22-08603-s001.zip › Supplemental materials_Analysis of PPARg signaling activity in psoriasis/Pathway models/Models images and html files/PPARG inhibits phenotypes assotiated with psoriasis/239356.html]

type 2 diabetes


# Disease type 2 diabetes

|  |  |
| --- | --- |
| URN | urn:agi-meshdis:Diabetes%20Mellitus%2c%20Type%202 |
| Total Entities | 1 |
| Connectivity | 9592 |
| Name | type 2 diabetes |

---

|  |  |
| --- | --- |
| ChildConcepts | maturity-onset diabetes of young |

---

|  |  |
| --- | --- |
| Pathway | Proteins Involved in Diabetes Mellitus Type 2 |
|  | PPARG inhibits phenotypes assotiated with psoriasis |
|  | efects selected |
|  | Neighbors of type 2 diabetes |
|  | Overview. Endocrine and metabolic diseases (chapter 4) |

---

|  |  |
| --- | --- |
| MedScan ID | 9002693 |

---

|  |  |
| --- | --- |
| Alias | Type II Diabetes |
|  | Diabetes Mellitus, Noninsulin-Dependent |
|  | Type 2 (insulin independent) diabetes |
|  | non insulin-dependent diabetic |
|  | Diabetes Type II |
|  | Ketosis-Resistant Diabetes Mellitus |
|  | Diabetes Type 2 |
|  | noninsulin dependent (Type 2) diabetes mellitus |
|  | Type 2 Diabetes Mellitus Non-Insulin Dependent |
|  | Adult-Onset Diabetes Mellitus |
|  | Stable Diabetes Mellitus |
|  | Ketosis Resistant Diabetes |
|  | Maturity-Onset Diabetes Mellitus |
|  | Type II Diabetic |
|  | non insulin-dependent diabetic mellitus |
|  | malnutrition-related diabetes mellitus |
|  | Type II Diabetes Mellitus |
|  | insulin-independent diabetes |
|  | malnutrition-related diabetes |
|  | Noninsulin-Dependent Diabetes Mellitus |
|  | non insulin-dependent diabetics |
|  | type 2 diabetics |
|  | Diabetes Mellitus, Adult-Onset |
|  | Diabetes Mellitus Type 2 |
|  | Diabetes Mellitus Type 02 |
|  | Diabetes, Adult Onset |
|  | Diabetes Mellitus, Maturity-Onset |
|  | non insulin dependent (Type 2) diabetes mellitus |
|  | Maturity-Onset Diabetes |
|  | insulin independent diabetes mellitus |
|  | Type 2 Diabetes Mellitus |
|  | fibrocalculous pancreatic diabetes |
|  | Diabetes Mellitus Type II |
|  | Diabetes Mellitus, Stable |
|  | TIIDM |
|  | fibro-calculous pancreatic diabetes |
|  | Type II Diabetes Mellitus Non-Insulin Dependent |
|  | non insulin dependent diabetes mellitus |
|  | noninsulin dependent diabetes |
|  | Adult-Onset Diabetes |
|  | noninsulin-dependent diabetic |
|  | noninsulin-dependent diabetic mellitus |
|  | noninsulin-dependent diabetics |
|  | NIDDM |
|  | Type 2 (insulin independent) diabetes mellitus |
|  | T2DM |
|  | Type 2 Diabetic |
|  | Diabetes, Maturity Onset |
|  | Type 2 Diabetes |
|  | Diabetes, Ketosis Resistant |
|  | maturity-onset diabetes of youth |
|  | noninsulin dependant diabetes mellitus |
|  | maturity-onset diabetes of the young, type 1 |
|  | maturity-onset type diabetes of young people |
|  | MODY |
|  | maturity-onset diabetes mellitus of the young |
|  | maturity-onset diabetes of the young, type 3 |
|  | MODY2 |
|  | maturity-onset diabetes of the young, type 2 |
|  | MODY5 |
|  | maturity-onset diabetes of young |
|  | maturity-onset diabetes of the young, type 5 |
|  | MODY3 |
|  | MODY1 |
|  | non insulin dependant diabetes mellitus |

---

|  |  |
| --- | --- |
| MeSH ID | D003924 |
|  | M0006155 |

---

|  |  |
| --- | --- |
| MeSH Heading | Diabetes Mellitus, Type 2 |

---
